# Liraglutide decreases carotid intima-media thickness in patients with type 2 diabetes: 8-month prospective pilot study

**DOI:** 10.1186/1475-2840-13-49

**Published:** 2014-02-22

**Authors:** Manfredi Rizzo, Manisha Chandalia, Angelo Maria Patti, Vittoria Di Bartolo, Ali A Rizvi, Giuseppe Montalto, Nicola Abate

**Affiliations:** 1 Biomedical Department of Internal Medicine and Medical Specialties, University of Palermo, Palermo, Italy; 2 Euro-Mediterranean Institute of Science and Technology, Palermo, Italy; 3 Division of Endocrinology, Diabetes and Metabolism, University of South Carolina School of Medicine, Columbia, South Carolina, USA; 4 Division of Endocrinology, The University of Texas Medical Branch, 8.138 Medical Research Building, 301 University Boulevard, 77555-1060 Galveston, TX, USA

**Keywords:** Liraglutide, Carotid intima-media thickness, Cardiovascular risk, Type2 diabetes

## Abstract

**Background:**

Liraglutide, a long-acting glucagon-like peptide-1 (GLP-1) analog, has several non- glycemic properties, but its effect on carotid intima-media thickness (IMT), a recognized marker of subclinical atherosclerosis, is still unknown.

**Methods:**

A prospective study of 8 months duration in 64 patients with type-2 diabetes and no prior history of coronary artery disease evaluated whether adding liraglutide to metformin affects carotid IMT, measured by color doppler ultrasound.

**Results:**

After 8 months, fasting glucose decreased by 2.1 mmol/l and HbA1c by 1.9% (p < 0.01 for all). Liraglutide reduced total-cholesterol and triglycerides by 10%, and LDL-cholesterol by 19%, whereas HDL-cholesterol increased by 18% (p < 0.01 for all lipid changes). Carotid IMT decreased from 1.19 ± 0.47 to 0.94 ± 0.21 mm (p < 0.01). Yet, changes in carotid IMT did not correlate with changes in any other variable studied.

**Conclusions:**

Liraglutide decreases carotid IMT after 8 months treatment independently of its effect on plasma glucose and lipids concentrations.

**Trial registration:**

ClinicalTrials.gov: NCT01715428.

## Introduction

Liraglutide is a long-acting human glucagon-like peptide-1 (GLP-1) analog suitable for once-daily administration in patients with type-2 diabetes (T2DM). Clinical studies have demonstrated glucose-reducing effects, improvements in pancreatic beta cell function and a low risk of hypoglycaemic events with this agent [1]. Liraglutide has also shown favorable effects on inflammatory markers, such as C-reactive protein (CRP), tumour necrosis factor-α (TNF-α) and plasminogen activator inhibitor type-1 (PAI-1) [2,3]. Further, liraglutide seems to also have an impact on diabetic dyslipidemia, reducing total cholesterol, triglycerides and low density lipoproteins (LDL)-cholesterol levels, with a concomitant increase in high density lipoproteins (HDL)-cholesterol [3,4].

These favorable effects of liraglutide on multiple metabolic pathways may have beneficial effects on atherosclerosis and possibly reduce risk for cardiovascular disease. However, prospective studies to elucidate the clinical impact of liraglutide on cardiovascular outcomes in patients with T2DM, such as the international LEADER (Liraglutide Effect and Action in Diabetes: Evaluation of Cardiovascular Outcome Results) study, are still ongoing [5]. We performed a pilot study to explore, for the first time, the effects of liraglutide on carotid intima-media thickness (IMT), a recognized marker of subclinical atherosclerosis, thus providing a preliminary view on the potential impact of this therapeutic intervention on cardiovascular risk of patients with T2DM.

## Methods

### Patients and methods

We studied 64 subjects with known or newly diagnosed T2DM (32 men and 32 women, mean age: 63 ± 8 years) who were consecutively referred to our Unit of Diabetes and Cardiovascular Prevention for a clinical evaluation. All subjects were on metformin therapy (doses ranging from 1500 to 2000 mg daily). The project design included a medical examination, biochemical analyses and the eco-color-doppler examination of carotid arteries. The procedures adopted were in agreement with the Helsinki Declaration of 1975 as revised in 1983, and were approved by the Ethics Council of the University of Palermo. This study has been registered in clinicaltrials.gov (NCT01715428).

All patients gave informed consent to participate in the study. At admission they underwent a medical examination and were excluded from the study if they had clinical evidence of liver dysfunction or renal failure. Waist circumference, height and weight were recorded, and BMI was calculated as kg/m^2^. Fifty-three percent of the subjects were current smokers and 64% had hypertension diagnosed by the following: systolic or diastolic blood pressure, respectively, ≥140 or ≥90 mmHg or previous pharmacological therapy with antihypertensive drugs. Forty-five patients were already taking anti-hypertensive or lipid-lowering drugs, and such medications were maintained at the same doses until the end of the study. In details, 21 subjects were taking beta-blockers, 15 angiotensin-converting enzyme inhibitors, 12 calcium entry blockers and 20 diuretics. Twenty-six patients were on statins, 9 on fibrates and 12 on omega-3 fatty acids. Eight patients were taking aspirin. None of the subjects included in the study had a previous major cardiovascular event (see Table 1).

**Table 1:** **Patients**’ **baseline characteristics (n = 64)**

Age (years)	63 ± 8
Women, n (%)	32 (50)
Diabetes duration (years)	9 ± 8
Smoking habit, n (%)	34 (53)
Past history of cardiovascular diseases, n (%)	0 (0)
Family history of cardiovascular diseases, n (%)	26 (41)
Systolic blood pressure (mmHg)	128 ± 13
Diastolic blood pressure (mmHg)	76 ± 6
Hypertension, n (%)	41 (64)
Dyslipidemia, n (%)	42 (66)
Obesity, n (%)	28 (44)
Diabetic complications, n (%)	16 (25)
Use of anti-hypertensive therapies	
Beta-blockers, n (%)	21 (33)
Angiotensin-converting enzyme inhibitors, n (%)	15 (23)
Calcium entry blockers, n (%)	12 (23)
Diuretics, n (%)	20 (31)
Use of lipid-lowering drugs	
Statins, n (%)	26 (41)
Fibrates, n (%)	9 (14)
Omega-3 fatty acids, n (%)	12 (19)
Aspirin use, n (%)	8 (13)

Metformin was continued at a fixed dose of 1500 mg daily, and liraglutide was added subcutaneously at a dose of 0.6 mg daily for the first 2 weeks, followed by 1.2 mg daily. Patients were followed up for 8 months.

### Biochemical analyses

At baseline and after 4 months and 8 months of therapy, serum glucose and HbA1c were measured after a 14-hour overnight fast. Total cholesterol, triglycerides and HDL-cholesterol were measured by standard enzymatic-colorimetric methods [6-8], while LDL-cholesterol was calculated using the Friedewald formula.

### Color doppler ultrasound of carotid arteries

B-mode real-time ultrasound was performed at baseline and after 4 and 8 months of therapy to evaluate the arterial wall thickness in the carotid arteries. All the examinations were performed by a single examinator (A.M.P.) using a single sonographer (Medison SonoAce Pico, with a probe of 7.5-10.0 MHz) in a blinded manner; it means that the examinator did not have access to previous scans when follow-up studies were performed. The ultrasound examination was performed in a standardized manner with fixed angles of insonation.

As previously reported [9], patients were examined in the supine position, and each carotid wall or segment was examined to identify the thickest intimal-medial site. Each scan of the common carotid artery began just above the clavicle, and the transducer was moved to the carotid bifurcation and along the internal carotid artery. Three segments were identified and measured in anterior and posterior planes on each side: the distal 1.0 cm of the common carotid artery proximal to the bifurcation, the bifurcation itself, and the proximal 1.0 cm of the internal carotid artery. At each of these sites we determined the IMT, defined as the distance between the echogenic line representing the intimal blood interface and the outer echogenic line representing the adventitial junction. The maximum carotid IMT value was used for analysis and determined as the mean of the maximum IMT of near- and far-wall measurements of both the left and right side arteries for each of the 3 arterial segments.

### Statistical analysis

Statistical analysis was performed using Statview^®^ 5.0 (SAS Institute Inc., Cary, NC, USA). Univariate analysis was performed using paired t-test, while correlation analysis was performed using the Spearman rank correlation method. ANOVA was used to test changes in all evaluated parameters from baseline to 4 months and to 8 months of treatment.

## Results

All patients took the prescribed medications without discontinuation. Two of the patients developed mild nausea but continued liraglutide throughout the study. As shown in Table 2, from baseline to 4 and 8 months of treatment, carotid IMT significantly decreased from 1.19 ± 0.47 to 1.04± 0.35 to 0.94 ± 0.21 (p = 0.0010), while there was a non significant reduction in body weight (from 81 ± 17 to 78 ± 16 to 78 ± 16 Kg, p = 0.44) and waist circumference (from 105 ± 14 to 102 ± 13 to 102 ± 13 cm, p = 0.36). By contrast, there was a significant decrease in fasting glucose (from 8.9 ± 3.8 to 7.1 ± 1.6 to 6.8 ± 1.9 mmol/L, p < 0.0001) and hemoglobin A1c (from 8.4 ± 0.8 to 6.7 ± 0.9 to 6.5 ± 0.8%, p < 0.0001).

**Table 2:** Effects of 8-month liraglutide therapy (n = 64)

	**Baseline**	**After 4 months**	**After 8 months**	***p*** **=**
				**(*for trend *)**
Weight (kg)	81 ± 17	78 ± 16*	78 ± 16*§	*0.44*
BMI (kg/m^2^)	30 ± 5	29 ± 5*	29 ± 5*§	*0.28*
Waist circumference (cm)	105 ± 14	102 ± 13*	102 ± 13*	*0.36*
Fasting glicemia (mmol/L)	8.9 ± 3.8	7.1 ± 1.6*	6.8 ± 1.9*	<*0.0001*
HbA1c (%)	8.4 ± 0.8	6.7 ± 0.9*	6.5 ± 0.8*	<*0.0001*
Total cholesterol (mmol/L)	4.4 ± 0.9	4.0 ± 0.7*	3.9 ± 0.7*	*0.0020*
Triglycerides (mmol/L)	1.8 ± 0.7	1.6 ± 0.7*	1.6 ± 0.7*	*0.26*
HDL-cholesterol (mmol/L)	1.07 ± 0.3	1.09 ± 0.3	1.16 ± 0.3*§	*0.19*
LDL-cholesterol (mmol/L)	2.5 ± 0.9	2.2 ± 0.6*	2.0 ± 0.7*	*0.0011*
Carotid IMT (mm)	1.19 ± 0.47	1.04 ± 0.35*	0.94 ± 0.21*§	*0.0010*

*p < 0.01 vs baseline; §p < 0.01 vs. 4 months.

Therefore (Table 2), after 8 months of therapy average body weight decreased by 3 kg, fasting glucose by 2.1 mmol/l, and HbA1c by 1.9% (p < 0.01 for all). Liraglutide also significantly reduced total-cholesterol and triglycerides by 10%, and LDL-cholesterol by 19%, with a concomitant 8% increase in HDL-cholesterol (p < 0.01 for all). Carotid IMT decreased by 0.25 mm after 8 months of therapy (-21% vs baseline). Yet, no significant correlation was found between changes in carotid IMT and changes in all the evaluated parameters (data not shown).

We have further performed multivariate analysis, in order to assess potential independent associations between decreased carotid IMT and clinical and laboratory variables evaluated at baseline. Such baseline parameters included gender, age, smoking habit, past history of cardiovascular diseases, duration of diabetes, as well as plasma HbA1c, LDL-cholesterol, HDL-cholesterol and triglycerides. We found an independent predective role for LDL-cholesterol (p = 0.0320) but not for any other variable examined.

We also investigated the relationship between decreased carotid IMT and the use of cardiovascular medications, including beta-blockers, angiotensin-converting enzyme inhibitors, calcium entry blockers, diuretics, statins, fibrates omega-3 fatty acids and aspirin (data not shown). Yet, we did not find any significant effect for any of the above-mentioned drugs. Finally, when the effect of liraglutide on carotid IMT was adjusted for the use of lipid-lowering drugs, the effect of liraglutide remained significant (data not shown).

## Discussion

This pilot study shows, for the first time, that liraglutide has desirable effect on carotid IMT in patients with T2DM after 8 months of therapy. This effect seems to be achieved by mechanisms independent of induced changes in plasma glucose and lipid concentrations. This is somewhat consistent to a very recent study, where GLP-1 analogue therapy reduced several inflammatory markers, independently of the glycaemic or body weight effects induced by the GLP-1 analogue [10]. Pioglitazone therapy also slowed progression of carotid atherosclerosis in subjects with prediabetes, independently of changes in cardio-metabolic risk factors [11].

Although not yet directly demonstrated, it is possible that liraglutide has beneficial effects on the process of atherosclerotic plaque formation. Pre-clinical data have shown that liraglutide is able to reduce infarct size in mice with experimental myocardial infarction [12], while exenatide, a synthetic GLP-1 receptor agonist, reduced intimal hyperplasia in insulin resistant rats [13]. Further, in patients with type-2 diabetes, the stimulation of GLP-1 secretion seems to have a significant impact on cardiovascular risk [14]. Yet, in a recent study a 6-month GLP-1 receptor agnist treatment did not modulate vascular function, despite significant improvements in body composition and glycaemic control [15].

Regarding other liraglutide effects found in the present study, body weight significantly reduced after 4 months of therapy but we found no further change after 8 months of treatment. In another study [16], liraglutide induced significant and persistent weight loss up to 6 months of follow-up in obese Japanese patients with type 2 diabetes. In the present study liraglutide treatment was also able to significantly reduce plasma total- and LDL-cholesterol concentrations; further, LDL-cholesterol was the only variable showing an independent associations with decreased carotid IMT at multivariate analysis. Yet, in a recent study performed in subjects with type-2 diabetes [17], apolipoprotein B and not LDL-cholesterol was able to predict subclinical carotid atherosclerosis.

Further, a large number of our patients were under cardiovascular drugs; yet, we did not find any significant relationship between the use of cardiovascular medications and carotid IMT. This finding may be potentially explained by: a) the relative small numbers of patients receiving each individual class of drugs; b) the very common use in our patients of multiple cardiovascular therapies, which therefore limits the identification of the effect of a specific class of drug.

Our findings on the beneficial effect of liraglutide on carotid IMT may be linked to the improved endothelial function by incretin-based therapies, and GLP-1 in particular (as reviewed in [18]). Indeed, using different methodologies, it has been shown that GLP-1 improves endothelial function in patients with type-2 diabetes or the metabolic syndrome; such methodologies included strain-gauged plethysmography and flow-mediated dilation [19-21]. Further, in pre-clinical studies, liraglutide improved endothelial function and inhibited the progression of vascular disease via the effects on plaque stability and endothelial function [22,23].

Previous studies have investigated the effects of anti-diabetic agents on carotid IMT. For instance, the CHICAGO (Carotid Intima-Media Thickness in Atherosclerosis Using Pioglitazone) trial [24] was a randomized, double-blind, multicenter study, which evaluated the effect of pioglitazone vs glimepiride on changes in carotid IMT in patients with type-2 diabetes. In this study, after 18 months of treatment, the change from baseline in carotid IMT (e.g., the prespecified primary end point) was -0.001 mm in the pioglitazone group and +0.012 mm in the glimepiride group. By contrast, in a very recent study [25], vildagliptin was able to reduce carotid IMT by about 0.10 mm after only 3 months of therapy in 90 patients with type-2 diabetes. This finding is somewhat consistent to our finding of a reduction of 0.15 mm after 4 months of liraglutide therapy.

Although our findings regarding changes in carotid IMT are of considerable interest, the impact of liraglutide on clinical cardiovascular outcomes is unknown, and further studies are needed to investigate its athero-metabolic actions. The very recent data on the cardiovascular outcome with the use of saxagliptin and alogliptin highlight the need of more studies for incretin-based therapies [26,27], including the GLP-1 analogues. It is hoped that the ongoing international LEADER study will provide useful answers in this regard [5].

A limitation of the present study is the lack of the placebo (control) arm. Such control group had to include patients undergoing metformin therapy only, e.g. without liraglutide. Although we did not investigate the effect of metformin alone on carotid IMT, previous studies have shown that metformin therapy has mild or null effects on carotid IMT [28-30]. It is therefore unlikely that metformin was responsible for the strong and significant decrease in carotid IMT that we have found after 4 and 8 months of therapy. Also, it needs to be highighted that in the present study we administered liraglutide as add-on therapy in patients who were already receiving metformin therapy. Finally, the statistical analysis revealed that the effect of liraglutide on several parameters (including carotid IMT) was very strong and highly significant; therefore, the probabilty of the occurrence of our findings by chance alone is extremely low.

Strengths of the study include the blinded measurements of carotid intima-media thickness as well as the excellent adherence to treatment. To our knowledge, the present study is the first one to examine the effects of liraglutide on carotid IMT.

## Competing interests

MR has given talks, attended conferences and participated in advisory boards and trials sponsored by Novo-Nordisk. NA is in the speakers bureau for Merck and Astra-Zeneca. MC is in the speakers bureau for Merck, Astra-Zeneca and Bristol-Myers Squibb. The other authors have no relevant conflict of interest to disclose.

## Authors’ contributions

MR wrote the manuscript and researched data. MC contributed to the discussion and reviewed/edited the manuscript. AP researched data and reviewed/edited the manuscript. VDB researched data. AAR contributed to the discussion and reviewed/edited the manuscript. GM wrote the manuscript and researched data. NA contributed to the discussion and reviewed/edited the manuscript. The guarantor for this work is MR Preliminary data on this study were reported at the 2012 Annual Conference of the American Diabetes Association. All authors read and approved the final manuscript.
